# Use of Cariprazine in emotional (bipolar) disorders

**DOI:** 10.1192/j.eurpsy.2025.1058

**Published:** 2025-08-26

**Authors:** C. Istikoglou, E. Andreou, N. Lampraki, I. Rizavas, M. Zisimou, G. Mavridis, D. Mousdi, M. Papazisi, E. Karameri, N. Georgiou, A. Ballas

**Affiliations:** 1 DEPARTMENT OF PSYCHIATRY, “KONSTANTOPOULEIO”-PATISION GENERAL HOSPITAL OF NEA IONIA; 2 DEPARTMENT OF PSYCHIATRY, PSYCHIATRIC HOSPITAL OF ATTICA, ATHENS, GREECE

## Abstract

**Introduction:**

Cariprazine is indicated for the treatment of Schizophrenia in adult patients. Its therapeutic action is dual: as a blocker of D2 dopamine receptors, as a partial agonist at D2 and D3 dopamine receptors, and as a partial antagonist at 5HT2B and 5HT2A receptors of serotonin, as well as a blocker of histamine H1 receptors.

**Objectives:**

The objective of this paper is to highlight the treatment of Emotional Disorders.

**Methods:**

30 patients (n=30) of the Outpatient Psychiatric Clinics of the Department of Psychiatry were studied, of which 15 men and 15 women, who suffered from Bipolar Disorder Type I. All patients were given the YMRS (Young Mania Related Scale) questionnaire, before starting treatment and 30 days after starting treatment with Cariprazine 6 mg, in combination with sodium valproate 1000 mg daily.

**Results:**

Of the 30 patients, 25 presented a clear decrease in the YMRS Scale (percentage 75%), of which 13 were women and 12 were men, respectively. In the present study no superiority in response to treatment was found for either men or women.

**Conclusions:**

Cariprazine is a new therapy in the treatment of Bipolar Disorder Type I. It is of course necessary to complete the study with a larger pool of patients.

**Disclosure of Interest:**

None Declared

